# A molecular recombination map of *Antirrhinum majus*

**DOI:** 10.1186/1471-2229-10-275

**Published:** 2010-12-15

**Authors:** Zsuzsanna Schwarz-Sommer, Thomas Gübitz, Julia Weiss, Perla Gómez-di-Marco, Luciana Delgado-Benarroch, Andrew Hudson, Marcos Egea-Cortines

**Affiliations:** 1 Max-Planck-Institut für Züchtungsforschung, Carl-von-Linné-Weg 10, 50829 Köln, Germany; 2 Deutsche Forschungsgemeinschaft (DFG)-Wissenschaftliche Geräte und Informationstechnik, D-53170 Bonn, Germany; 3 Institute of Plant Biotechnology (IBV), Technical University of Cartagena, Campus Muralla del Mar, 30202 Cartagena, Spain; 4 Instituto de Botánica del Nordeste (IBONE)- CONICET-Facultad de Ciencias. Agrarias, Universidad Nacional del Nordeste (UNNE) CC 209, Corrientes 3400 Argentina; 5 Institute of Molecular Plant Sciences, University of Edinbugh,, King's Buildings, Mayfield Rd., Edinburgh EH9 3JH, UK

## Abstract

**Background:**

Genetic recombination maps provide important frameworks for comparative genomics, identifying gene functions, assembling genome sequences and for breeding. The molecular recombination map currently available for the model eudicot *Antirrhinum majus *is the result of a cross with *Antirrhinum molle*, limiting its usefulness within *A. majus*.

**Results:**

We created a molecular linkage map of *A*. *majus *based on segregation of markers in the F2 population of two inbred lab strains of *A. majus*. The resulting map consisted of over 300 markers in eight linkage groups, which could be aligned with a classical recombination map and the *A. majus *karyotype. The distribution of recombination frequencies and distorted transmission of parental alleles differed from those of a previous inter-species hybrid. The differences varied in magnitude and direction between chromosomes, suggesting that they had multiple causes. The map, which covered an estimated of 95% of the genome with an average interval of 2 cM, was used to analyze the distribution of a newly discovered family of MITE transposons and tested for its utility in positioning seven mutations that affect aspects of plant size.

**Conclusions:**

The current map has an estimated interval of 1.28 Mb between markers. It shows a lower level of transmission ratio distortion and a longer length than the previous inter-species map, making it potentially more useful. The molecular recombination map further indicates that the *IDLE *MITE transposons are distributed throughout the genome and are relatively stable. The map proved effective in mapping classical morphological mutations of *A. majus*.

## Background

*Antirrhinum majus*, the garden snapdragon, has been used as a model system for genetics since the early 20^th ^Century [1]. It is a member of a monophyletic group of about twenty five species that are native to the Mediterranean region share the same chromosome number (2n = 16) and are able to form fertile hybrids with each other [2]. The majority of species are allogamous, though cultivated *A. majus *lines and a few other wild species can self-fertilize.

A collection of *A*. *majus *mutants has been produced from some laboratory lines of *A. majus *selected for high transposon activity [3]. In several cases, these have been used to clone the corresponding genes by transposon tagging (e.g. [4-10]). In addition there is a collection of roughly four hundred classical mutants, mostly in an isogenic background (Sippe 50) [11,12]. The majority of these mutants does not show the genetic instability characteristic of transposon-induced mutations, and therefore have limited use for transposon tagging. The alternative approach of gene isolation by positional cloning is currently restricted by the availability of molecular recombination maps in *Antirrhinum*, though it has recently been successful in isolating the *fistulata *(*fis*) gene [13]. Though a classical *fis *mutation was genetically stable, it is caused by insertion of a miniature inverted-repeat transposable element (MITE), which is present in relatively low copy-number in all *Antirrhinum *species. Because the transposon family appeared relatively inactive it was called *IDLE*.

The existing molecular recombination map for *Antirrhinum *was built using the F2 of a cross between *A. majus *(line 165E) and a wild relative, *A. molle *[14]. The map identified eight linkage groups and use of common loci had allowed these to be related to a classical genetic map and to the *A. majus *chromosomes by in situ hybridization [15]. However, the majority of markers from the *A. molle *x *A. majus *hybrid showed significantly distorted transmission, which are likely to have affected the accuracy of the map, and the map also contained clusters of loci consistent with chromosome rearrangements between the species [12,15]. Such rearrangements were also suggested by observation of chromosomes [15]. These two factors would hinder attempts to map *A. majus *mutations in crosses to *A. molle*. A further disadvantage of using inter-species crosses to map *A. majus *mutations is that *A. majus *and *A. molle *differ in many morphological characters, including plant and organ size. Segregation of natural variation would therefore be likely to obscure the effects of mutations in hybrid mapping populations.

We therefore developed a linkage map of *A. majus *using the inbred lines Sippe50 and 165E. The map consists of 302 markers (protein coding sequences, AFLP and transposons), covering nearly 95% of the genome. As a proof of concept, we placed on the map six mutations affecting floral and overall size. We also mapped the distribution of *IDLE *transposons, revealing that they are allocated with coding genes in all *Antirrhinum *chromosomes.

## Results & discussion

### Construction of a molecular linkage map for *A. majus*

To construct a molecular linkage map for *A. majus *we crossed two inbred lines, 165E and Sippe 50. Line 165E originated from cultivated *A. majus *in the UK and is phenotypically distinct from Sippe 50, which was derived in Germany, possibly from a wild accession [11,16]. A single F1 progeny was self-pollinated to produce an F2 mapping population of 96 plants. This population therefore contains 192 recombinant copies of each chromosome, sufficient for mapping loci to a resolution of ~ 1 cM. The F2 population was genotyped at 377 loci. These included 90 protein-coding genes, in which polymorphisms were detected by sequencing the alleles from both parents. The identities of the protein-coding genes are given in Table 1. The remaining markers mainly comprised AFLP and insertions of the MITE transposon *IDLE *[13]. The genotype data were used to estimate a recombination map. Fifty-nine AFLP markers (28% of the total) and one dominant *IDLE *insertion were present in significantly more or fewer F2 plants than expected and could either not be mapped or mapped only by reducing support for linkage groups significantly. These markers were therefore rejected. The remaining markers formed a map comprising 90 protein-coding genes, 87 of which were mapped as co-dominant CAPS or size polymorphisms, 159 dominant AFLP and 53 *IDLE *insertions (10 with co-dominant alleles and 43 dominant markers). A complete list of primers for each marker and the corresponding map position can be found in Additional file 1. At nine loci AFLP bands from both parents showed complete linkage in repulsion and were subsequently treated as synthetic co-dominant markers.

**Table 1:** List of EST-based markers and functional annotation.

Sequence number	EST annotation	Length in bp	min. eValue
EM:AMA558924	thioredoxin peroxidase	723	1.0E-1.12106E-85
EM:AJ801757	proline-rich apg-like protein	670	1.0E-2.50815E-86
EM:AJ794773	o-linked c-transferase	758	1.0E-2.06855E-101
EM:AJ804794	histone h1	556	1.0E-1.27731E-27
EM:AJ794598	at4g20410-like protein	736	1.0E-1.80515E-107
EM:AJ805499	stearoyl-acyl carrier protein desaturase	481	1.0E-9.70457E-56
EM:X57295	TAP1 protein precursor	5280	1.0E-1.98821E-44
EM:AY072736	HIRZINA KNOX protein	1462	1.0E-0.0
EM:AJ802708	Eukaryotic translation initiation factor 6	630	1.0E-1.16373E-87
EM:AJ794444	mgc108135 protein	675	1.0E-6.11236E-80
EM:AJ802293	translational inhibitor protein	589	1.0E-1.37912E-65
EM:AJ803243	60 s ribosomal protein l18	731	1.0E-2.80747E-92
EM:AJ559267	heavy-metal-associated domain-containing protein	504	1.0E-2.98463E-13
EM:AJ790658	urease accessory protein g	561	1.0E-3.52407E-97
EM:AJ792971	gtp-binding protein	628	1.0E-1.89892E-82
EM:AJ560201	iron-sulfur cluster assembly protein	768	1.0E-3.45238E-59
EM:AJ620906	STYLOSA co-repressor	2865	1.0E-0.0
EM:AJ806659	atp-dependent clp protease proteolytic subunit	711	1.0E-1.54692E-116
EM:AJ560074	leucine rich repeat protein	754	1.0E-2.04947E-101
EM:AM422773	YABBY4 transcription factor	1395	1.0E-7.9976E-177
EM:X97639	cyclin-dependent kinase, CDC2a	1038	1.0E-2.78255E-170
EM:AJ806654	C2H2 zinc-finger protein	530	1.0E-4.79832E-31
EM:AM422772	YABBY2 transcription factor	1373	1.0E-3.22687E-114
EM:AJ568130	cyclic nucleotide-regulated ion channel	620	1.0E-3.55048E-94
EM:AJ568031	amino acid permease familyexpressed	1459	1.0E-1.38642E-78
EM:AJ800340	at3g04780 f7o18_27	721	1.0E-1.77924E-75
EM:AJ568062	chloroplast translation initiation factor 2	415	1.0E-4.51282E-45
EM:S53900	PLENA MADS-box transcription factor	1073	1.0E-1.61386E-120
EM:AJ801384	actin associated protein	538	1.0E-5.43416E-33
EM:AJ800042	multiple stress-responsive zinc-finger protein	781	1.0E-1.45489E-60
EM:AJ805889	centromere microtubule binding protein cbf5	692	1.0E-3.39584E-97
EM:AJ808934	transcription factor	884	1.0E-2.17962E-88
EM:AJ568063	polygalacturonase-inhibiting protein	734	1.0E-1.19729E-66
EM:AJ804237	histidinol dehydrogenase	634	1.0E-2.00488E-95
EM:AJ795662	flavonoid 3-o-glucosyltransferase	750	1.0E-9.60942E-83
EM:AJ800415	erwinia induced protein 2	615	1.0E-2.67616E-49
EM:AJ796551	Frigida	666	1.0E-5.06947E-39
EM:AJ796122	p-p-bond-hydrolysis-driven protein transmembrane transporter	747	1.0E-3.07514E-73
EM:AJ800197	atp-dependent protease clp atpase subunit	594	1.0E-3.10775E-69
EM:AJ559052	t-complex protein 1 epsilon tcp-1-	690	1.0E-2.62699E-116
EM:AJ803361	monodehydroascorbate reductase	730	1.0E-7.35389E-109
EM:AJ802640	isochorismatase hydrolase	779	1.0E-1.51824E-94
EM:AY223518	LIP1 apetala2-like transcription factor	1845	1.0E-0.0
EM:AJ805150	photoassimilate-responsive protein par-like protein	530	1.0E-7.5899E-61
EM:AJ620905	STYLOSA1 co-repressor	3123	1.0E-0.0
EM:AJ801986	at-rich element binding factor 3	590	1.0E-8.38628E-55
EM:X68831	GLOBOSA MADS-box transcription factor	6108	1.0E-5.69779E-27
EM:X76995	polygalacturonase-inhibiting protein	3545	1.0E-4.66003E-119
EM:AJ558819	psap psi-p ptac8 tmp14 (thylakoid membrane phosphoprotein of 14 kda) dna binding	762	1.0E-2.07028E-40
EM:AJ793550	trna-methyltransferase subunit	535	1.0E-7.89349E-61
EM:AJ620909	SEUSS3A co-repressor	3683	1.0E-0.0
EM:AJ800998	Transcription factor lim	710	1.0E-3.7918E-99
EM:AJ620910	seu3b protein	2004	1.0E-2.36396E-82
EM:AY451399	CRABSCLAW-like YABBY transcription factor	732	1.0E-1.78657E-70
EM:AJ794665	nuclear cap binding protein subunit 2	673	1.0E-1.25817E-85
EM:AJ802365	---NA---	583	1.0E-1.68821E-23
EM:AJ801224	---NA---	613	1.0E-2.33555E-13
EM:AJ794216	nuclear RNA binding	758	1.0E-1.84607E-33
EM:AJ794472	serine threonine protein kinase	746	1.0E-1.35578E-75
EM:AJ568099	pgr5-like a	1478	1.0E-9.46041E-75
EM:AJ791655	bzo2h3 (arabidopsis thaliana basic leucine zipper 63) dna binding transcription factor	758	1.0E-9.80266E-27
EM:AJ790549	immunophilin	566	1.0E-1.28799E-54
EM:M55525	FLORICAULA transcription factor	1545	1.0E-0.0
EM:AJ802861	---NA---	728	1.0E-1.22273E-47
EM:AJ799233	delta-12 oleate desaturase	736	1.0E-8.2567E-116
EM:AJ804300	protein	683	1.0E-6.22658E-88
EM:AJ803800	af319475_1 alpha-expansin 9 precursor	590	1.0E-5.56035E-91
EM:AJ803115	protein	654	1.0E-9.83593E-72
EM:AJ790136	tfIIb-related protein	677	1.0E-1.59963E-96
EM:AJ805759	eukaryotic translation initiation factor 6	689	1.0E-1.55443E-110
EM:AJ559280	tic20-like protein	710	1.0E-6.95853E-77
EM:AJ620907	SEUSS1 protein	3495	1.0E-0.0
EM:AJ789733	---NA---	675	1.0E-6.54566E-74
EM:AJ800463	tubulin alpha-6 chain	605	1.0E-4.42202E-40
EM:AJ791186	Glutaredoxin-like protein	684	1.0E-1.57483E-30
EM:AJ790836	b12 d protein	564	1.0E-1.21222E-36
EM:AJ794452	duf1230-containing protein	763	1.0E-1.97117E-91
EM:AJ793362	phosducin-like protein 3	578	1.0E-6.36625E-60
EM:Y16313	CYCLOIDEA TCP transcription factor	861	1.0E-1.34443E-129
EM:AY223519	LIP2 apetala2-like transcription factor	1914	1.0E-0.0
EM:AJ800866	polycomb protein ez1	507	1.0E-1.11628E-31
EM:AJ805209	rer1a protein	582	1.0E-1.72796E-60
EM:AJ568117	phenylalanine ammonia-lyase	619	1.0E-7.46548E-60
EM:AJ791765	microtubule motor	745	1.0E-7.82606E-45
EM:AJ808258	flu (fluorescent in blue light) binding	663	1.0E-2.25085E-39
EM:AJ793379	rpm1-interacting protein 4	580	1.0E-1.07793E-22

The ESTs were automatically annotated using BLAST2GO. The corresponding EST annotation was performed using a minimal threshold of e-6. Those genes with high homology to genes with unknown function were annotated as NA. Sequences with known mutant phenotypes in *Antirrhinum *are given their *Antirrhinum *gene names.

**Figure 1:**
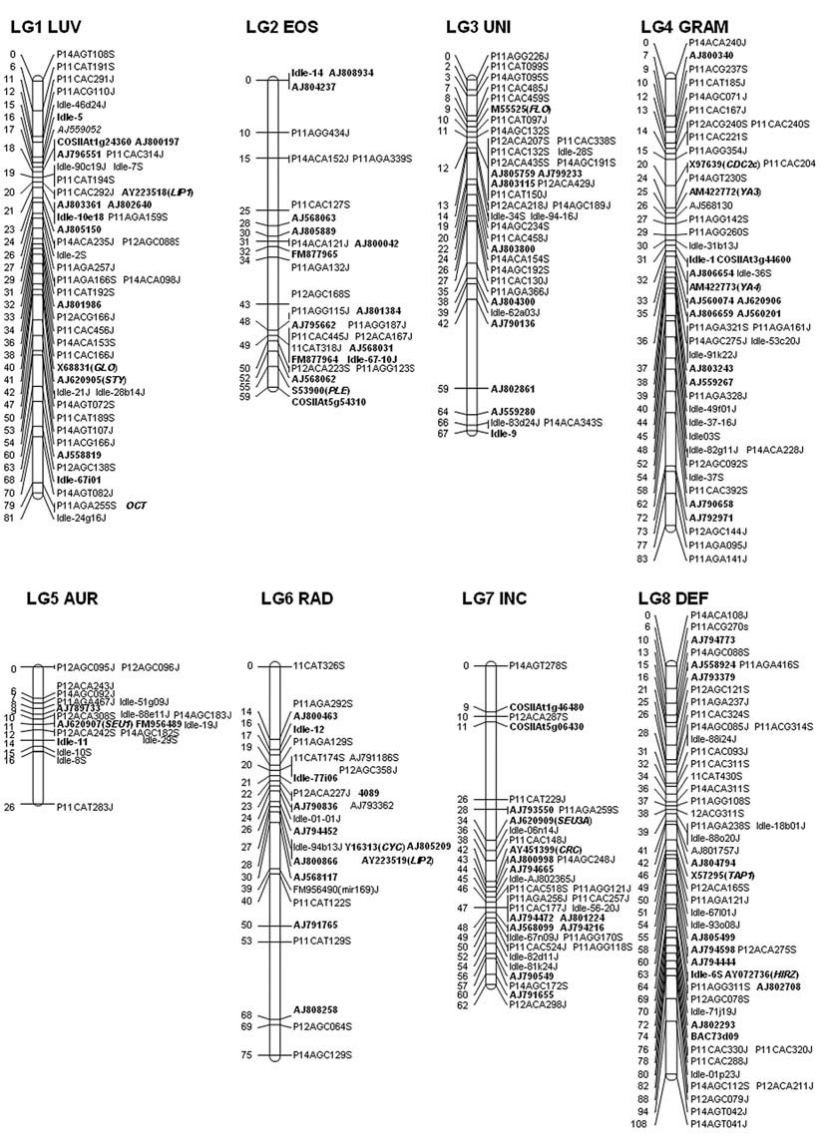
**A molecular linkage map for *Antirrhinum majus *Sippe 50 × 165E**. The eight linkage groups are oriented, numbered and named as in previous *Antirrhinum *maps. Positions are given in centiMorgans (Kosambi). Protein-coding loci are named with their EMBL accession numbers as in Table 1 and with their *Antirrhinum *gene names in italics, where their functions are known from mutants. *IDLE *denotes a locus carrying an insertion of an *IDLE *transposon in one of the parents and loci with the suffix P are AFLP (see Materials & Methods for AFLP nomenclature). Loci with co-dominant alleles are shown in bold and those with dominant alleles in regular type.

The resulting map comprised eight linkage groups with a total length of 562 cM that was estimated to cover 95% of the genome (Figure 1). At this level of coverage, the average interval between markers was 2.0 cM, with 88% of the genome estimated to lie within 2.0 cM of a marker and 99% within 5 cM. Although the average interval between co-dominant markers was 6.0 cM, a similar proportion of the genome (83%) was within 2.0 cM of the nearest co-dominant marker. Assuming a haploid genome size of 3.6 × 10^8 ^bp for *A. majus *[17], a marker interval of 2.0 cM represents on average 1.28 Mbp of DNA.

### Map comparison

A previous molecular map for *Antirrhinum *had been produced from the F2 progeny of a cross between *A. majus *(line 165E) and the wild species *A. molle *[14]. To allow identification and alignment of linkage groups in the two populations, the genotypes from the previous mapping population were used to reconstruct a map using the same parameters as for the *A. majus *x *A. majus *F2. Markers common to both maps allowed identification of corresponding linkage groups and their orientations (Additional file 2).

**Figure 2:**
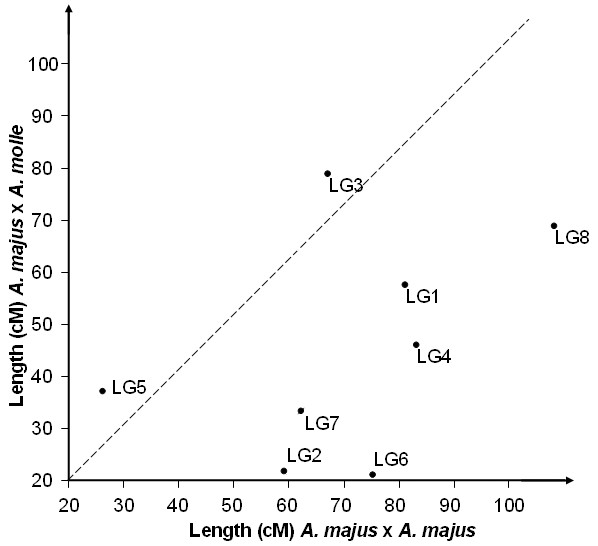
**Comparative genetic lengths of chromosomes in the *A. majus *x *A. molle *and *A. majus *x *A. majus *maps**. The estimated lengths of each of the eight linkage groups in the two maps are plotted against each other.

The total *A. majus *map was about 54% larger than for *A. majus *x *A. molle*. However the variations in length differed in magnitude and direction between chromosomes (Figure 2). Two linkage groups (5 and 3) were slightly smaller in map A, while the remainders were significantly longer, suggesting that the causes of length differences varied between chromosomes.

Previous studies have reported both smaller and larger maps for intra-specific crosses as compared to inter-specific crosses [18,19]. Several factors might contribute to variations in map lengths for *Antirrhinum *and might differ between chromosomes. Of particular relevance to the utility of the *A. majus *map is the possibility that the two marker sets cover different parts of the genome. However, this seems unlikely, because although the two maps contain a different number of loci (296 in map A and 227 in map B) they are estimated to cover a similar proportion of the genome (95% in A and 94% in B using Method 4 [20].) Moreover, randomly removing 69 markers from A, to make the numbers of markers the same in both populations, reduced the length of map A by an average of only 2% (Additional file 3). Similarly maps made only with dominant protein-coding genes from each F2 population showed the same trends in map length differences (data not shown), suggesting that they are not dependent on the number or types of markers used.

The insensitivity of map length to the type of marker also suggests that the 51 mapped *IDLE *transposons were relatively stable, because excision of an *IDLE *in members of the mapping population would result in the wrong parental origin being assigned to its locus and an over-estimation of recombination frequencies. The relative stability of *IDLE *markers was further supported by the finding that they were no more likely than other marker types to have an apparent recombination breakpoint immediately next to them, as would be expected if excision had resulted in an incorrect genotype.

Although many transposon families are predominant components of heterochromatin, MITE transposons have commonly been found associated with gene-rich regions [21,22]. This is consistent with the observed distribution of *IDLE *insertions in *Antirrhinum*, which are interspersed with protein-coding genes and do not appear to be clustered in centromeric or telomeric regions.

### Transmission ratio distortion differences between inter and intra-specific maps

**Figure 3:**
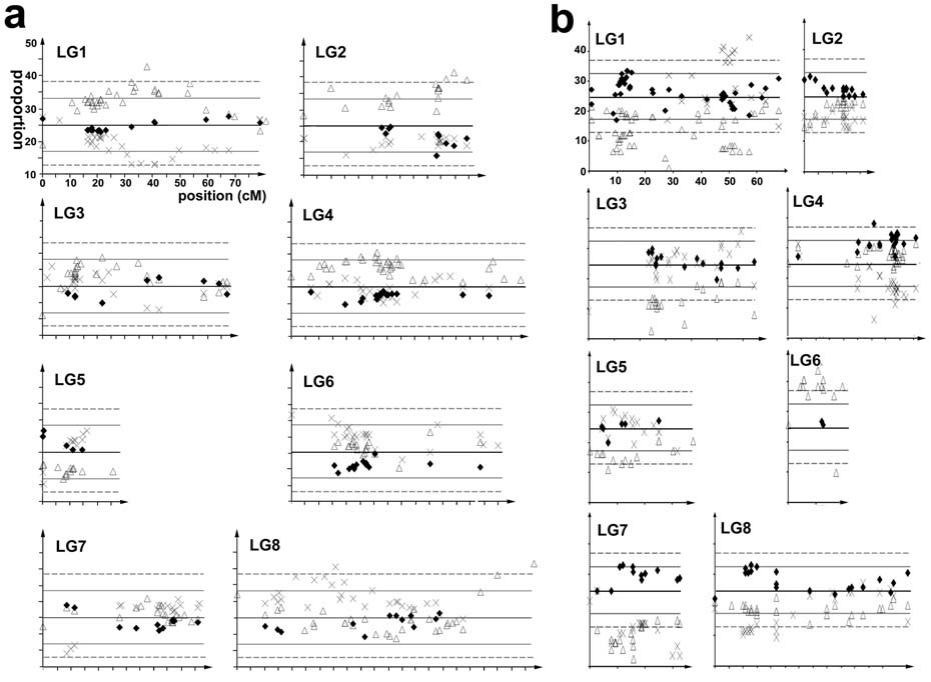
**Transmission of parental alleles to F2 mapping populations**. For the *A. majus *x *A. majus *population (a) the proportion of Sippe 50 homozygotes (crosses), 165E homozygotes (triangles) and half the proportion of heterozygotes (diamonds) is plotted for each locus according to its position in the eight linkage groups (LG). The solid horizontal line represents the expected average proportion (0.25) of each genotype class that is expected in the absence of distorted transmission. The solid and broken grey lines represent the approximate thresholds for significantly distorted genotype frequencies at the 0.95 and 0.99 levels, respectively. Genotype frequencies for the *A. majus *x *A. molle *population are shown in (b). The genotype frequencies and significance levels are represented as in a), except that crosses denote *A. molle *homozygotes.

At least some of the length variation between maps might be attributed to transmission ratio distortion (TRD). This was more marked in the interspecies cross, in which loci representing most of the genome deviated significantly from their expected Mendelian ratios (Figure 3). It was most severe for LG6, in which *A. molle *carries a functional gametophytic self-incompatibility (*S*) locus. This prevented recovery of F2 plants homozygous for *A. molle *alleles at LG6 unless recombination had occurred between the marker and the *S *locus. In contrast, *A. majus *lacks a functional *S *locus and shows only mildly distorted transmission of markers from LG6 (Figure 3). TRD can lead to under-estimation of map distances [23] and loss of marker information, for example no dominant markers closely linked to the *A. molle S *allele were identified in map B. It can also lead to markers being wrongly assigned to linkage groups. The lower level of TRD in map A therefore justifies the use of mapping populations of *A. majus *rather than inter-species hybrids.

It was previously suggested that the clustering of markers in map B may be caused by chromosome inversions that distinguished *A. majus *from *A. molle *[14], preventing mapping of loci that lie within inversions. However, there is significant clustering (p < 0.0001) of markers in both maps and significantly more clustering in map A than map B. Since fewer rearrangements are expected between two *A. majus *lines than between *A. majus *and *A. molle *clusters of markers appear unlikely to represent inversions.

### Map validation by mapping mutations affecting size

**Figure 4:**
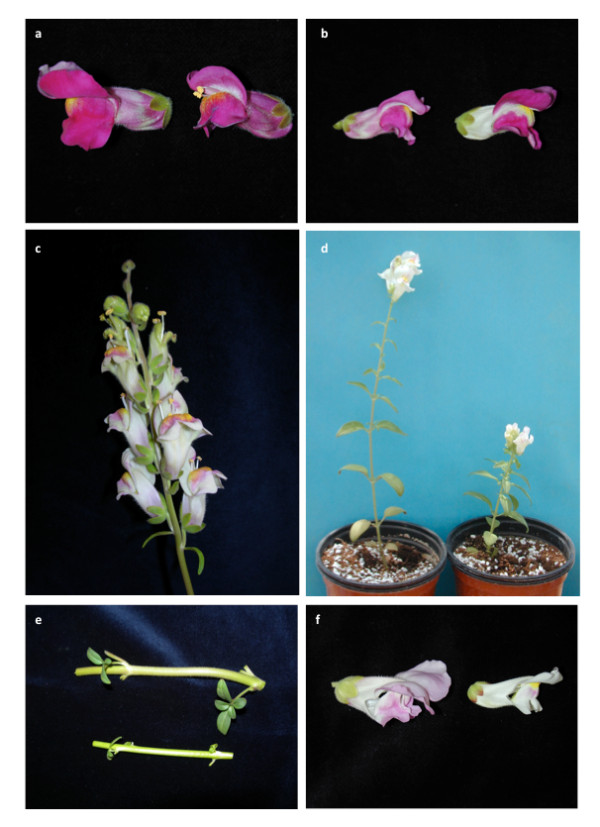
**Phenotypic characteristics of the mutants used to validate the map**. Phenotypes of *compacta *(a), *formosa *(b), *compacta ähnlich *(c) and *nana *(d), Pictures show wild type on the left and mutant on the right. The mutant *heroina *(e) above and wild type below. Stems of *hero *correspond to same internode in siblings. The mutant *Nitida *(f) wild type left, mutant right.

One possible use of a molecular map is to determine the chromosomal positions of loci that have been identified by mutation. This can, for example, classify mutations that are potentially allelic, which is particularly useful for dominant mutations, and allows isolation of the affected genes on the basis of their positions. We therefore tested the utility of the *A. majus *map in determining the position of six classical mutations affecting aspects of plant size (Figure 4). All six mutations were in the Sippe 50 mutant background and therefore crossed to wild-type 165E to generate F2 mapping populations.

The *nana *(*na*) mutation described at the end of the XIXth century in the Vilmorin catalog [24], reduces plant size in a recessive fashion and flowers early irrespective of photoperiod. The *na *mutant phenotype segregated as expected for a recessive mutation in the F2 generation of the cross to 165E. However, a second allele *nana^largiflora^*, which caused a somewhat weaker phenotype in the Sippe 50 genetic background [12], could not be distinguished from wild-type in F2 populations produced by crossing to 165E line. This highlights a potential problem arising from suppression of a weak mutant phenotype in a cross between two lines that differ, albeit slightly, in morphology. Another difficulty was identified in the case of the recessive *muscoides *(*mus*) mutation, which causes dwarfism. No *mus *mutants were initially identified in the F2 of the cross with 165E. However, *mus *mutants were recovered at a low frequency (2 out of 60 plants) when F2 seeds were germinated in Petri dishes, suggesting that the *mus *mutation can be lethal in the 165E genetic background.

The mutant *hero *affected stem thickness, a trait that seems to be partly controlled by genes affecting floral size in Arabidopsis like *Bigbrother *and *Kluh *[25,26]. However *hero *did not show a statistical difference from wild-type in lateral organ size, either in the original Sippe 50 background or in F2 populations, and segregated as expected of a recessive mutation in the F2 (data not shown).

Four mutations affecting floral size also segregated as expected in the F2 populations produced by crossing to 165E with *compacta *(*co*)*, compacta-ähnlich *(*coan*) and *formosa *(*fo*) mutations appearing fully recessive, and *Nitida *(*Ni*) as semi-dominant [11]. In the case of *Ni *and *co *mutants, their phenotypes in the F2 were similar to those of the original background while *coan*, and *fo *mutants showed slightly larger differences from wild-type.

The mutants affect floral size in different ways, *coan *decreases flower size without affecting vegetative body size [27], while the *co *mutation reduces both flower and lateral organ size. The *fo *mutation increases floral size, [28] while the *na *mutation reduces plant height and leaf width without significantly affecting flower size while the *Ni *mutation reduces the sizes of flowers, leaves and internodes in a dosage-dependent fashion.

As an initial approach, the mutations were mapped by bulk-segregant analysis [29]. DNA was extracted from several pools of four plants that shared the same phenotype and screened with a CAPS marker located in a middle region of each chromosome arm (a total of 16 markers). Markers that χ^2 ^tests suggested were not linked to the size mutation were rejected. Where evidence for linkage was found, additional CAPS markers from the same chromosome regions were used to analyze individual F2 plants to refine map positions. Statistically significant linkage was found between the additional markers and the mutations in all cases (Table 2). The distance between a mutation and the closet marker ranged from the *coan *locus and the marker AJ790136 in LG3, which showed no recombination in 43 homozygous mutants, to *na *and AJ568062 in LG2 which were separated by about 26 cM.

**Table 2:** Map position of six mutants.

Mutant	Closest Marker	LG	Marker position (cM)	Kosambi distance (cM)	n	significance
***coan***	AJ790136	**LG3**	FLO	0	43	*
***co***	AJ568117	**LG6**	30	3.1	49	*
***Fo***	*SEU3A*	**LG7**	34	7.2	21	*
***hero***	*SEU3A*	**LG7**	34	4.6	22	*
***na***	AJ568062	**LG2**	52	25.9	21	*
***Ni***	*PLE *(S53900)	**LG2**	55	9.1	50	*

Mutants were originally obtained in the Sippe 50 background and mapped by crossing with 165E. Markers were considered significantly linked for χ^2 ^tests of p < 0.05

We have therefore shown that it is feasible to map mutations in crosses between these two *Antirrhinum *lines, even mutations with relatively subtle effects on plant size. Extending this approach to map based cloning should become more feasible as the density of molecular markers in *Antirrhinum *increases. However, the ability to map with even moderate resolution can be used to identify potentially allelic mutations, including natural variants. One of the major features of *Antirrhinum *species is that they differ widely from each other in size. Several genes underlying this size variation have been mapped as quantitative trait loci (QTL), e.g. [30]. Like the size mutants analyzed here, the QTL can affect a single type of organ or have more pleiotropic effects. It should now be possible to identify whether any classical size mutations might correspond to size QTL on the basis of map positions and so select candidate mutations for more allelism tests. A corresponding classical mutation can facilitate QTL isolation and the understanding of QTL gene function.

## Conclusions

We have constructed a molecular linkage map using two inbred lines of *Antirrhinum majus*, 165E and Sippe 50. The newly developed map has eight linkage groups and a total length of 562 cM with an estimated coverage of 95% of the genome. There is an average interval of 2 cM between codominant markers in 88% of cases and 5 cM in 99%, and assuming a genome size of 3.6 × 10^8 ^bp, an interval of 2 cM represents on average 1.28 Mbp of DNA.

The new map is 54% longer than the previously published map of *A.majus *x *A. molle*, and this difference is caused by increased length of the different linkage groups, except 3 and 5 that were slightly shorter indicating that map length differences were the result of differences between chromosomes in the two crosses.

We have mapped 51 IDLE transposons that are interspersed with EST-based markers indicating that MITE transposons, like in other plants, are found in gene-rich regions. Determination of EST-based markers will allow future use of the *A*.*majus *map for comparative genomic studies with other plants.

The new map has fewer regions of TRD reinforcing its usefulness to determine genome positions with higher accuracy. This has been achieved by validating the map with six classic mutants affecting floral size (*Ni, co, coah *and *fo*), body size (*Hero *and *na*) and flowering time (*na*). We have been able to obtain map positions for each mutant using F2 mapping populations.

## Methods

### Plant material

Seeds of *Antirrhinum majus *L. were germinated and grown as described by [31].

The *A. majus *line Sippe 50 [11] was obtained originally from the IPK Gatersleben and maintained by self-pollination while the second wild-type line 165E was produced by several generations of self-pollination from line JI.98 [16,32]. An F2 population (*n *= 96) for mapping molecular markers was selected at random from the progeny of a single F1 hybrid of Sippe 50 × 165E. The mutants *compacta *(*co*) [33], *compacta ähnlich *(*coan*) [12], *formosa *(*fo*), *Grandiflora *(*Graf*), *heroina *(*Hero*), *Nitida *(*Ni*) [11] and *nana *(*na*) [24] were obtained from the IPK Gatersleben collection. All the mutants are in the Sippe 50 genetic background. Mutations were mapped in F2 populations produced by crossing mutants to the 165E wild-type.

DNA was extracted using a NucleoSpin^® ^kit (Macherey-Nagel) from 100 mg of frozen leaf samples that had been ground to a powder in liquid N_2_.

### Mapping transcribed genes

Sequence tagged sites (STS) were generated using primers able to amplify regions from a collection of *A. majus *EST sequences that showed differences between Sippe50 and 165E [34]. The identities of PCR products were confirmed by sequencing. For six genes, both parental alleles could be distinguished by differences in the sizes of their amplified products in agarose gels without digestion. A further three loci amplified from only one parent and were therefore treated as dominant markers. For the remaining genes, restriction site polymorphisms were identified by comparing sequences of amplified products and the loci scored as co-dominant CAPS resolved in agarose gels. The ESTs used to develop markers were annotated automatically using the BLAST2GO program [35,36].

### AFLP analysis

AFLP were amplified from DNA that had been digested with *Pst*I and *Mse*I using eight combinations of selective primers. Primers for the *Pst*I ends of fragments had 3' selective di-nucleotides AA (P11), AC (P12) or AT (P14) and were labeled with one of four different fluorescent dyes (6-FAM, VIC, NED or PET) while those at the *Mse*I ends had 3' extensions of ACA, AGC, AGT, CAC or CAT. Products were separated with the LIZ-500 internal size standard (ABI) using an ABI 3730 DNA Analyzer. Output files were converted to fsa format using the program obtained from http://dna.biotech.wisc.edu/ABRF/3730toGSconverter.exe, processed using Genescan software (ABI) and the presence or absence of bands scored from virtual gels created using a version of the program Genographer http://hordeum.oscs.montana.edu/genographer/ that had been modified by its authors to accommodate the five different color channels. AFLPs were scored as dominant markers. They were named according to the primers used to generate them, their size and their parent of origin - e.g. locus 11AGA141J amplified with selective primers P11 and Mse-AGA as a band of 141 nt and originated from parent 165E.

### Mapping MITE transposons

Different copies of the *IDLE *transposon were identified by homology to the insertion in the *fistulata-2 *mutation in *A. majus *[13] either by hybridization to genomic clones or comparison to *A. majus *BAC clones. The host sequences to both sides of 10 *IDLE *insertions were identified. In these cases, primers from the two flanking regions were used to distinguish the presence or absence of the transposon on the basis of size polymorphism allowing these loci to be scored as co-dominant markers. For 43 insertions only one flanking sequence was obtained and a flanking primer was used with an *IDLE *primer to detect the presence of an insertion, which was treated as a dominant marker.

### Map construction

To construct the molecular recombination map for the F2 population, co-dominant markers were scored as one of three allelic states (homozygous 165E, homozygous Sippe 50 or heterozygous) while dominant markers were assigned to one or other parent and scored for the presence or absence in F2 individuals. A map was estimated from the genotype data at 377 loci using Joinmap 3.0 [37], using a minimum LOD score of 6.0 to identify potential linkage groups. Maps of each linkage group were then established using the default thresholds for elimination of markers and establishing marker order and the Kosambi mapping function [38] to calculate genetic distances. Transmission ratio distortion was represented for loci with co-dominant alleles by plotting the frequencies of each homozygote and half the frequency of heterozygotes and for dominant loci by the frequency of homozygotes lacking the dominant allele. Each class was expected with a frequency of 0.25 and significant deviations from this expectation were assessed with χ^2 ^tests.

Total genome size was estimated using Method 4 from [20] or by adding twice the average marker spacing to each chromosome, with both methods providing very similar estimates. The percentage of the genome within a particular map distance of the nearest molecular marker was estimated with the method used by [39]. To analyze whether markers showed non-random clustering, the number of 1 cM intervals expected to contain a particular number of markers was calculated from the total number of markers and map length, assuming that the markers were distributed randomly (i.e. that the number of markers per 1 cM interval followed a Poisson distribution). This null hypothesis was tested against the observed frequency distribution of markers, using a χ^2 ^test. The frequency distributions of marker densities for the two maps were also compared directly, using a χ^2 ^test.

### Mutant mapping

To map mutations, F2 plants were selected for genotyping on the basis of their phenotype. Four pools, each containing a similar amount of DNA from four homozygous plants, were first used to scan for linkage to one of 16 markers-representing both arms of all eight chromosomes. Linkage to a marker locus was suggested by an enrichment of one of its parental alleles in more than one of the pools (i.e. enrichment of the Sippe 50 allele in pools of recessive mutations or the 165E allele in wild-type pools in the case of dominant mutants). Suspected linkage was investigated further by genotyping between 20 and 60 F2 individuals for the original locus and at additional loci linked to it. Linkage was assessed using χ^2 ^tests to identify significant deviations from random segregation in the mutant population and the Kosambi function used to estimate map distances between mutations and markers from recombination frequencies.

## List of abbreviations

AFLP: Amplified fragment length polymorphism; cM: centiMorgan; *Co: Compacta; Coan Compacta ähnlich; Fo: Formosa; Hero: Heroina*; MITE: Miniature Inverted Repeat Transposable element; *Na: Nana; Ni: Nitida*; TRD: transmission ratio distortion

## Authors' contributions

ZsSS, TG, AH and MEC designed experiments. ZsSS developed the EST and *IDLE *markers, TG and AH did the AFLP markers, TG, ZsSS and AH made the map. MEC did the bioinformatic analysis of EST annotation. PGC, LDG and JW mapped the mutants. PGC, LDG, JW and MEC grew the F2 populations and scored the phenotypes. MEC did the mutant pictures. LDG, JW and MEC did the phenotypic analysis of the mutants. AW and MEC wrote the draft and all the authors except ZsSS read, corrected and approved it.

## Supplementary Material

Additional file 1 **Map positions, primers and restriction enzymes used to detect *IDLE *transposons and EST-based markers**. Click here for file

Additional file 2 **Anchoring of linkage groups in map B (on the left) to those of the newly created map A (right)**. Click here for file

Additional file 3 **The effects of marker number on the length of map A. Map B contained 69 fewer markers than map A**. To investigate whether a larger number of markers was responsible for the longer length of map A, 69 markers were removed at random and map A recalculated. This was repeated 1,000 times with removal of a different set of randomly selected markers each time. The frequency distribution of total map lengths obtained in the simulations is shown. The average length was 552 cM, a reduction of only 2% from the map estimated with all markers. Therefore a larger number of markers does not account for map A being 54% longer than map B Click here for file
