# Supplementary figures and images for: A molecular recombination map of Antirrhinum majus
Source: BMC Plant Biol. 2010 Dec 15;10:275. doi: 10.1186/1471-2229-10-275 (PMC3017841; doi:10.1186/1471-2229-10-275)

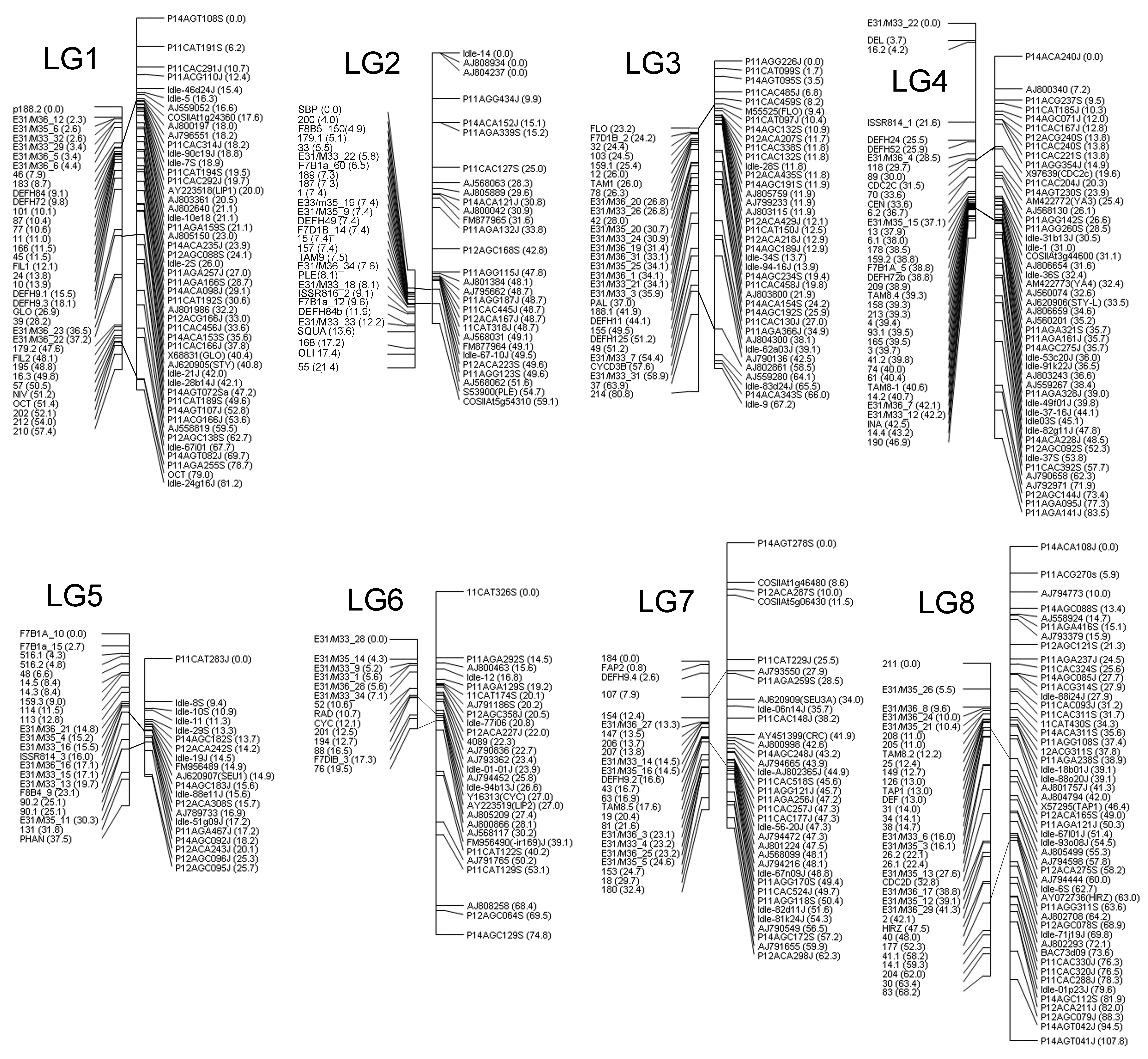

Supplement: Additional file 2 — Anchoring of linkage groups in map B (on the left) to those of the newly created map A (right). [file 1471-2229-10-275-S2.PNG]

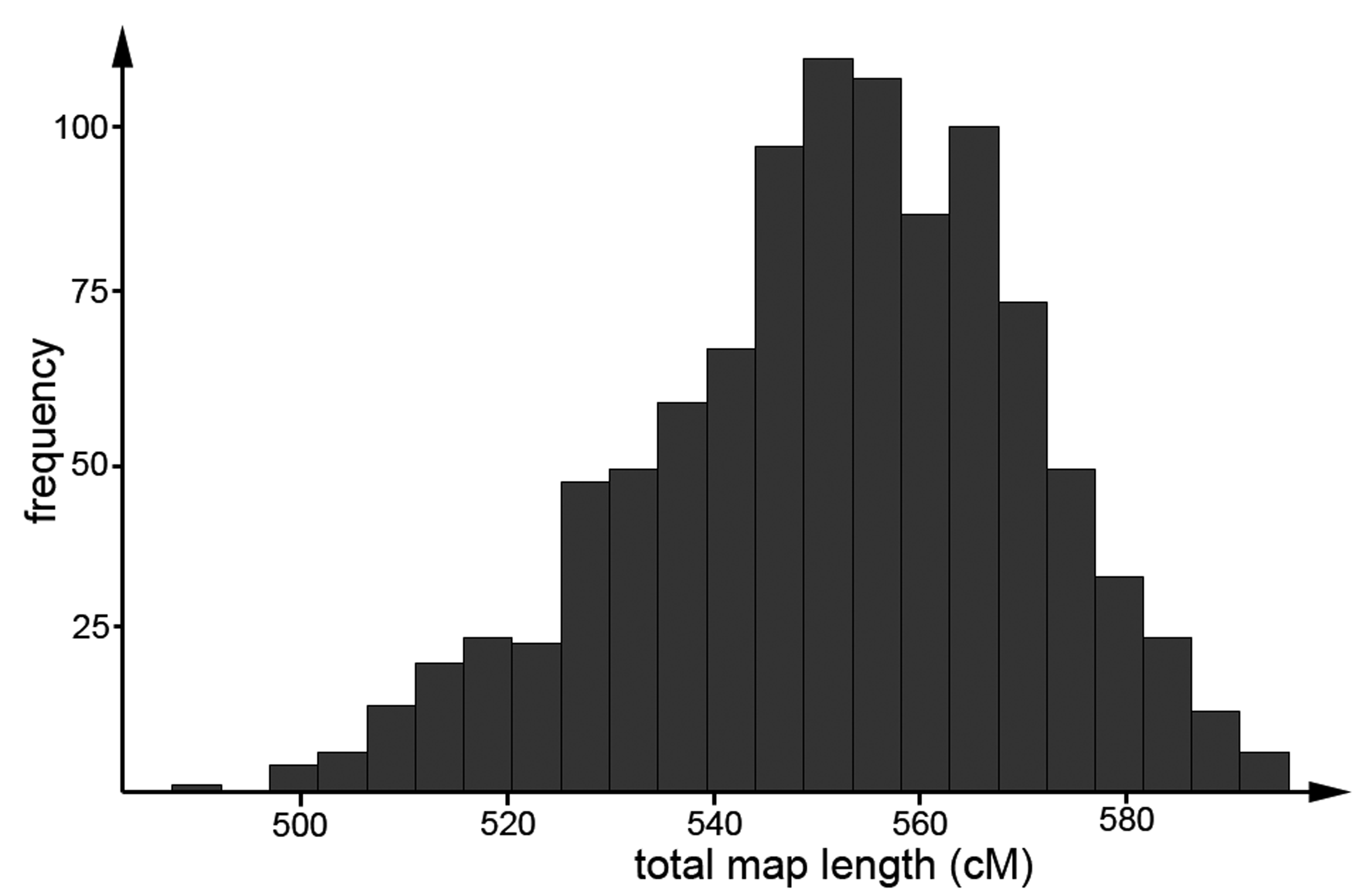

Supplement: Additional file 3 — The effects of marker number on the length of map A. Map B contained 69 fewer markers than map A. To investigate whether a larger number of markers was responsible for the longer length of map A, 69 markers were removed at random and map A recalculated. This was repeated 1,000 times with removal of a different set of randomly selected markers each time. The frequency distribution of total map lengths obtained in the simulations is shown. The average length was 552 cM, a reduction of only 2% from the map estimated with all markers. Therefore a larger number of markers does not account for map A being 54% longer than map B [file 1471-2229-10-275-S3.TIFF]
